# Influence of environmental factors on seed germination and seedling characteristics of perennial ryegrass (*Lolium perenne* L.)

**DOI:** 10.1038/s41598-022-13416-6

**Published:** 2022-06-09

**Authors:** Muhammad Mansoor Javaid, Athar Mahmood, Dalal S. Alshaya, Muneera D. F. AlKahtani, Hasnain Waheed, Allah Wasaya, Sher Aslam Khan, Maria Naqve, Imran Haider, Muhammad Adnan Shahid, Muhammad Ather Nadeem, Saira Azmat, Bilal Ahmad Khan, Rashad Mukhtar Balal, Kotb A. Attia, Sajid Fiaz

**Affiliations:** 1 Department of Agronomy, College of Agriculture, University of Sargodha, Sargodha, 40100 Pakistan; 2 Department of Agronomy, University of Agriculture Faisalabad, Faisalabad, 38040 Pakistan; 3 Department of Biology, College of Science, Princess Nourah Bint Abdulrahman University, P.O. Box 84428, Riyadh, 11671 Saudi Arabia; 4 College of Agriculture, Bahauddin Zakariya University, Bahadur Sub-Campus, Layyah, 31200 Pakistan; 5 Department of Plant Breeding and Genetics, The University of Haripur, Haripur, 22620 Pakistan; 6 Department of Botany, University of Agriculture Faisalabad, Faisalabad, 38040 Pakistan; 7 Horticultural Sciences Department, UF/IFAS, North Florida Research and Education Center Quincy 32351, University of Florida, Gainesville, USA; 8 Agriculture Extension and Adaptive Research, Agriculture Department, Government of Punjab, Lahore, Pakistan; 9 Department of Horticulture, College of Agriculture, University of Sargodha, Sargodha, 40100 Pakistan; 10 Center of Excellence in Biotechnology Research, King Saud University, P.O. Box 2455, Riyadh, 11451 Saudi Arabia

**Keywords:** Natural variation in plants, Plant physiology, Plant stress responses

## Abstract

Information regarding the germination and seedling growth behavior of a potential weed species is an important tool to manage weeds without the use of agricultural chemicals that cause harmful effects on human health and the environment. A series of experiments were directed to investigate the influence of different environmental factors (temperature, pH, NaCl, moisture stress, and seed burial depth) on germination and seedling emergence of perennial ryegrass (*Lolium perenne* L.) under controlled conditions. Results suggested that 25 °C is the optimum temperature for maximum germination (95%) and seedling growth of perennial ryegrass, however, a quick decline was observed at 35 °C. Seed germination was unaffected by pH levels ranging from 5 to 10. The 92% seed germination was recorded where no salt stress was applied and germination was reduced by 87% at 250 mMNaCl concentration. Seed germination was unaffected by osmotic potential ranges from 0 to − 0.4 MPa thereafter declined and completely inhibited at − 0.8 or − 1.0 MPa. No seed emerged at the soil surface or a soil depth of 6 or 7 cm and 90% emergence occurred at 1 cmsoil depth. The germination and seedlings parameters like time to initial germination, mean germination time, time taken to 50% germination and germination index, root and shoot length, and fresh and dry weight of root and shoot are significantly affected with the environmental factors. The information obtained in this study will be helpful to develop better management strategies for germination and the emergence of perennial ryegrass in areas where it has the ability to rapidly colonize.

## Introduction

Perennial ryegrass (*Lolium perenne* L.) is an important pasture and forage weed that often grows in temperate regions and it is native to Europe and Africa^1–3^. Perennial ryegrass has little or no juvenility and can be vernalized in the seed^4^ The Inflorescence of perennial ryegrass is un-branched; each spikelet has only one glume and 4 to 14 florets without awns. Though it was introduced by pastoralists as cultivated species for livestock grazing and fodder but from sown pastures, it has spread to occupy footpaths, roadsides, sand dunes, river beds, and waste places. In many parts of the world, the species is considered an environmental weed as it has many weedy characteristics like rapid adaptation to the environment, producing a large number of seeds, and is easily dispersed^5^. Due to high seed production and effective dispersal capacity, perennial ryegrass has the ability to rapidly colonize new areas where conditions are favorable.

The germination and emergence of weed seeds are two of the most critical phases in plant development and determine the success of a weed in an agro-ecosystem^6^. Germination and emergence are mediated by various environmental factors such as temperature^7^, pH^6^, NaCl^8^, water stress and seed burial depth^6^. Previous studies showed that initiation or inhibition of seed germination is dependent on these factors^9–11^. It is further claimed that temperature is a main determinant of germination when other factors are not limiting and temperature effects are variable for species within the genera^12^. Moisture stress may delay, reduce or prevent the weed seed germination however, the ability of weed seeds to germinate under low moisture conditions gives them a competitive advantage, which enables them to outcompete the germination or growth of many corps. The pH influenced the germination of seeds and availability of nutrients but several weed species tolerate a varied range of pH levels^13^. Weed seed location in the soil affected germination and emergence^6^, by affecting moisture, light and temperature^11^. According to Javaid et al.^14^ seed burial depth and food reserves are the most important factors affecting seed germination. Salinity is one of the main problems of soil degradation. Osmotic and ionic effects occur due to salinity that inhibits plant growth^15^. The seed germination affected by salinity depends upon the type of species and environmental characteristics^16^ and a reduction in germination was seen when the salt concentration exceeds a threshold level^10,17^. Salt stress generally decreases the germination percentage and retards the onset of germination. Overall germination of several species may be influenced more by low osmotic potential than by specific ion effects^18^. The specific ion effect strongly inhibited radicle growth and effective seedling formation depending on the frequency and the total rainfall to germinate and grow^19^.

A little work has been done on the germination ecology of this weed. The ecology information can play a key role in developing management or control programs, otherwise, control measures can be wasted. Therefore, the present research was planned to investigate the impact of temperature, soil pH, NaCl concentration, osmotic stress and seed burial depth on seed germination and seedling growth of perennial ryegrass. This information can help to characterize the germination niche and the habitat in which it is likely to germinate and develop.

## Materials and methods

### Site description and collection of seed

The experiments were performed in the Laboratory of Agronomy Department, College of Agriculture, University of Sargodha, Pakistan (32.0°N and 72.6°E) in 2018. Mature seeds were collected during March 2017 and 2018 from several fallow farms in Layyah, and Sargodha Punjab, Pakistan, and a bulked sample was prepared. At the time of collection, seeds were collected by breaking the stem of the plant about 10 cm in length with a spike each year separately. After that paper bags were used to transport the seeds into the laboratory, seeds were detached from the spike of the plant, cleaned, and air-dried at room temperature for 7 days.

Working samples were drawn from the composite sample. After that seeds were kept in air-tight glass bottles until used in the germination experiments.

### General germination test protocol

Before the commencement of subsequent germination or emergence tests, seeds of perennial ryegrass were sterilized in 1% sodium hypochlorite (NaClO) for 5 min and then rinsed with distilled water 5 times^20^. Germination of perennial ryegrass was determined by placing 20 seeds in a Petri plate of 9 cm diameter having a Whatman filter paper No. 10, moistened with 3 mL distilled water or the applicable treatment solution^14^. Para-film was used to seal the Petri plates to prevent the loss of water. The Petri plates were kept in a germination cabinet (Seedburo Equipment Company, Chicago, IL, USA). Cool white fluorescent bulbs (FL40SBR; National, Tokyo, Japan) were used to produce a photosynthetic photon flux density of 200 µmol m^−2^ s^−1^, set to a 12-h alternating light/dark cycle for all the experiments. All experimental trials were performed at the day and night temperature of 25 °C except for the temperature experiment. Seeds were conceived to be germinated when the radicle attained 2 mm in length. The germinated seeds were counted daily for 30 days. However, in the experiment of seed burial depths, when cotyledon was visible at the soil surface then seedlings were considered to have emerged. Each experiment was repeated twice using the seeds collected in two different years. Each treatment was replicated four times in each experiment.

### Impact of temperature

To investigate the impact of temperature on germination of perennial ryegrass, twenty seeds were placed uniformly in a Petri plate, lined with filter paper beneath the seed moistened with distilled water of 3 mL and then retained in an incubator at constant temperatures of 20, 25, 30 and 35 °C for 15 days.

### Impact of pH

The impact of pH on seed germination of perennial ryegrass was evaluated by using buffer solutions of pH 5, 6, 7, 8, 9 and 10 which were made according to the method defined by Chachalis and Reddy^21^. A 2 mM solution of MES [2-(*N*-morpholino) ethanesulfonic acid] was adjusted to pH 5 or 6 with 1 N hydrochloric acid (HCl), and a 2 mM solution of HEPES [*N*-(2-hydroxy-methyl) piperazine-*N*-(2-ethanesulfonic acid)] was adjusted to pH 7 or 8 with 1 N NaOH. Buffer solutions of pH 9 and 10 were prepared with 2 mM TRICINE [*N* Tris (hydroxymethyl) methylglycine] and adjusted to each respective pH value with 1 N NaOH. Unbuffered deionized water (pH 6.2) was used as a control.

### Impact of salt stress

To determine the influence of salt stress on seed germination of perennial ryegrass 20 seeds were placed in the sealed Petri dishes containing various sodium chloride (NaCl) concentrations at 0, 50, 100, 150, 200, 250, and 300 mM. However, distilled water was used as a control treatment.

### Impact of osmotic stress

Perennial ryegrass seeds were placed in Petri plates with the osmotic potential of 0, − 0.2, − 0.4, − 0.6, − 0.8 and − 1.0 MPa. Osmotic potentials were made by using polyethylene glycol (PEG 8000; Sigma-Aldrich Co., 3050, Spruce St., MO 63130) in distilled water. The equation described by Michel and Kaufmann^22^ was used for the calculation of water potential from a known concentration of PEG 6000. Distilled water was used as the control treatment.

Water potential = − (1.18 × 10^–2^) C − (1.18 × 10^–4^) C^2^ + (2.67 × 10^–4^) 18 CT + (8.39 × 10^–7^) C^2^ T. Where: T represents the temperature in centigrade while C is the PEG concentration.

### Impact of seed burial depth

The impact of seed burial depth on seed emergence was investigated in the greenhouse at the College of Agriculture, University of Sargodha, Pakistan. Twenty seeds of perennial ryegrass were placed on the soil surface or covered with soil (30% silt, 30% clay and 40% sand) at sowing depths of 0, 1, 2, 3, 4, 5, 6 and 7 in 15 cm diameter of plastic pots. In the entire experiment, the greenhouse temperature was maintained at 25 ± 2 °C during the day and night. Pots were watered as and when required to maintain sufficient soil moisture. Seedlings were considered to have emerged when cotyledons were visible at the soil surface.

Germination or emergence percentage data of perennial ryegrass obtained from the experiments regarding osmotic stress, NaCl concentration and seed burial depth, were subjected to non-linear regression analysis. Germination percentage data at various concentrations of osmotic potential and NaCl were fixed to a 3-parameter logistic model by using software Sigma Plot 2008 (version 11.0, SyStat Software GmbH, Schimmelbuschstrasse 25 D-40699 Erkrath Germany)). The fitted model was:

1 $$ G(\% ) = \frac{{\mathop G\nolimits_{\max } }}{{1 + \mathop {\left( {\frac{x}{{\mathop x\nolimits_{50} }}} \right)}\nolimits^{g} }} $$

where *G* is the total germination percentage at concentration *x*, *x*_50_ is the osmotic potential or NaCl concentration for 50% suppression of the maximum germination and *g* denotes the slope and *G*_*max*_ is the maximum germination percentage.

A three-parameter logistic model:

2 $$ E\left( \% \right) = \frac{{E_{max} }}{{1 + \left( {\frac{x}{{x_{50} }}} \right)^{e} }} $$

was fixed to the seedling emergence percentage gained at various burial depths of 0 to 7 cm, where *E* is the total seedling emergence percentage at burial depth *x*, *x*_50_ is the burial depth for 50% suppression of the maximum seedling emergence and *e* denotes the slope, *E*_*max*_ is the maximum seedling emergence percentage.

Time to initial germination or emergence (Ti) was noted when the first seed germinated or seedling emerged. The time taken to 50% germination or emergence (*T*_50_ or *E*_50_) was estimated by using a formula described by Coolbear et al.^23^:

3 $$ T_{50} \;or\;E_{50} = t_{i} \frac{{\left( {\frac{N}{2} - n_{i} } \right)(t_{j} - t_{i} )}}{{(n_{j} - n_{i} )}} $$

where *N* is the final number of sprouted or emerged seeds, and *n*_*j*_ and *n*_*i*_ are the cumulative number of seeds germinated by adjacent counts at times *t*_*j*_ (day) and *t*_*i*_, (day), respectively, when *n*_*i*_ < *N*/*2* < *n*_*j*._

Mean germination or emergence time (*MGT* or *MET*), which is a measure of the speed of germination or emergence, was calculated after Ellis and Roberts^24^:

4 $$ MGT\;or\;MET = \frac{{\sum {Dn} }}{\sum n } $$

where *n* is the number of seeds that had germinated on day *D* and *n* is the number of days counted from the beginning of the germination experiment.

The germination or emergence index (*GI* or *EI*), which is a measure of the percentage and rate of germination was calculated as described by the Association of Official Seed Analysis^25^ using the following formula:

5 $$ GI\;{\text{or}}\;EI = \frac{{No\;of\;germinated\;{\text{or}}\;emerged\;seedlings}}{Days\;of\;first\;count} + \cdots + { }\frac{{No\;of\;gerimnated\;{\text{or}}\;emerged\;seedlings}}{Days\;of\;final\;count} $$

Data regarding root length (cm), shoot length (cm), root fresh weight (g) root dry weight (g), shoot fresh weight (g) shoot dry weight of perennial ryegrass was collected throughout the study by using standard procedures.

### Statistical analysis

All the experiments were carried out in a completely randomized design (CRD) with four replications and each experiment was repeated twice. The collected data were subjected to one-way ANOVA. The significance of treatment means was practiced by using the least significant difference (LSD) test at a 5% level of probability^26^.

### Research ethics

Experimental research and field studies on plants (either cultivated or wild), including the collection of plant material, complied with relevant institutional, national, and international guidelines and legislation. Prior permission was undertaken from farm owner and Offices of Research, Innovation and Commercialization, University of Sargodha, Pakistan.

## Results

### Impact of temperature

**Figure 1:**
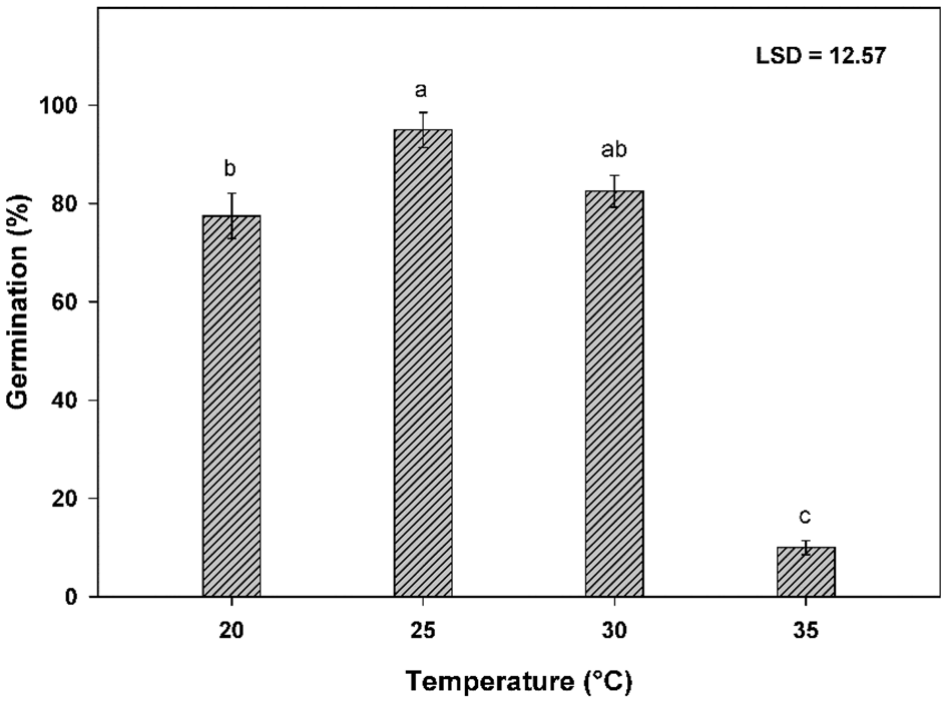
Influence of temperature on germination of perennial ryegrass. Nails on the vertical bars represent the standard error of the means.

Germination (%) of perennial ryegrass varied significantly in response to tested temperature (Fig. 1). The highest germination percentage of perennial ryegrass was observed at 25 °C. The germination at 20 and 30 °C temperatures were statistically similar. However, a strong reduction in germination was seen when the temperature was raised to 35 °C and reduces the germination by 85% compared to 25 °C.

The time to initial germination (Ti) of perennial ryegrass was 1 to 2 days earlier at 25 °C than other tested temperatures and was 2 days. The slowest Ti was measured at 30 °C (Table 1).

**Table 1:** Effect of temperature, pH, NaCl concentration, osmotic potential and seed burial depths on germination parameters of perennial ryegrass.

Treatments	Ti in days	T_50_ or E_50_ in days	MGT or MET in days	GI or EI
**Temperature °C**				
20	3^b^ (0.0)	5.3^a^ (0.64)	6.2^a^ (0.25)	2.9^b^ (0.23)
25	2^c^ (0.0)	2.4^b^ (0.01)	3.2^b^ (0.06)	6.5^a^ (0.26)
30	4^a^ (0.0)	5.8^a^ (0.52)	6.4^a^ (0.33)	2.8^b^ (0.13)
35	3.7^a^ (0.25)	3.5^b^ (0.28)	4.1^b^ (0.37)	0.5^c^ (0.04)
LSD at 0.05	0.38	1.38	0.87	0.56
**pH**				
Control (6.2)	2.7^b^ (0.25)	3.4^c^ (0.32)	4.1^c^ (0.35)	5.0^a^ (0.58)
5	3.0^ab^ (0.0)	4.9^b^ (0.37)	5.6^b^ (0.22)	3.6^bc^ (0.17)
6	3.2^a^ (0.25)	5.0^b^ (0.16)	5.4^b^ (0.09)	3.9^b^ (0.15)
7	2.7^ab^ (0.25)	5.0^b^ (0.20)	5.7^b^ (0.20)	3.6^bc^ (0.12)
8	3.0^ab^ (0.0)	6.2^a^ (0.30)	6.6^a^ (0.27)	3.0^c^ (0.18)
9	3.0^ab^ (0.0)	5.2^b^ (0.07)	5.8^b^ (0.08)	3.4^bc^ (0.03)
10	3.0^ab^ (0.0)	5.5^ab^ (0.23)	5.7^b^ (0.07)	3.5^bc^ (0.07)
LSD at 0.05	0.39	0.76	0.62	0.73
**NaCl concentration (mM)**				
Control	2.25^c^ (0.25)	2.6^d^ (0.10)	3.6^d^ (0.28)	5.6^a^ (0.44)
50	2.75^bc^ (0.25)	3.4^c^ (0.10)	4.1^cd^ (0.21)	4.35^b^ (0.28)
100	3.0^b^ (0.0)	3.7^c^ (0.04)	4.6^bc^ (0.04)	3.4^c^ (0.19)
150	3.0^b^ (0.0)	3.6^c^ (0.02)	4.4^c^ (0.07)	2.81^c^ (0.19)
200	3.2^b^ (0.25)	4.4^b^ (0.35)	5.0^b^ (0.18)	1.3^d^ (0.08)
250	7.5^a^ (0.28)	7.4^a^ (0.20)	8.0^a^ (0.14)	0.38^e^ (0.13)
300	NG	NG	NG	NG
LSD at 0.05	0.63	0.53	0.53	0.73
**Osmotic potential (MPa)**				
Control	2.2^c^ (0.25)	3.3^c^ (0.37)	4.0^c^ (0.30)	5.2^a^ (0.39)
-0.2	4.2^b^ (0.25)	5.7^b^ (0.20)	6.3^b^ (0.17)	2.5^b^ (0.08)
-0.4	4.7^b^ (0.25)	6.2^b^ (0.14)	7.1^b^ (0.11)	1.2^c^ (0.10)
-0.6	6.2^a^ (0.25)	8.0^a^ (0.35)	8.6^a^ (0.29)	0.6^c^ (0.05)
-0.8	NG	NG	NG	NG
-1.0	NG	NG	NG	NG
LSD at 0.05	0.77	0.88	0.73	0.63
**Seed burial depths (cm)**				
0	NG	NG	NG	NG
1	3.2^c^ (0.25)	4.9^d^ (0.28)	5.7^a^ (0.25)	3.5^a^ (0.24)
2	3.7^bc^ (0.25)	6.1^c^ (0.16)	6.7^b^ (0.03)	2.5^b^ (0.13)
3	4.5^b^ (0.28)	6.4^c^ (0.36)	7.0^c^ (0.08)	2.2^b^ (0.08)
4	5.2^b^ (0.25)	7.3^b^ (0.61)	8.0^c^ (0.41)	1.3^c^ (0.06)
5	7.2^a^ (0.25)	9.6^a^ (0.51)	10.2^d^ (0.27)	0.6^d^ (0.07)
6	NE	NE	NE	NE
7	NE	NE	NE	NE
LSD at 0.05	1.26	0.76	0.39	0.39

Ti, time to initial germination; T_50_ or E_50,_ time to obtain 50% germination or emergence; MGT or MET, mean germination or emergence time; GI, germination index; EI, emergence index; NG or NE, no germination or emergence. The values within the column followed by different letters were significantly different at *P* ≤ 0.05. LSD = Least significant difference. Values in parentheses are standard errors of the mean.

The minimum time to reach 50% of the maximum germination (*T*_50_) was recorded at 25 °C and the lowest mean germination time (*MGT*) was measured under the same temperature. The germination index (*GI*) represents the rate of germination and the highest *GI* was observed at 25 °C (Table 1). The seedlings' growth of perennial ryegrass was affected significantly by tested temperatures (Table 2).

**Table 2:** Effect of temperature, pH, NaCl concentration, osmotic potential and seed burial depths on seedling growth of perennial ryegrass.

Treatments	Root			Shoot		
	Length (cm)	Fresh weight (g)	Dry weight (g)	Length (cm)	Fresh weight (g)	Dry weight (g)
**Temperature °C**						
20	6.96^a^ (0.13)	0.08^b^ (0.01)	0.01^b^ (0.00)	8.52^ab^ (0.19)	0.54^ab^ (0.02)	0.07^a^ (0.00)
25	8.38^a^ (0.14)	0.13^a^ (0.00)	0.02^a^ (0.03)	9.31^a^ (0.27)	0.58^a^ (0.03)	0.08^a^ (0.04)
30	5.05^b^ (1.01)	0.07^b^ (0.01)	0.01^b^ (0.00)	7.39^b^ (0.67)	0.48^b^ (0.04)	0.06^a^ (0.06
35	3.42^c^ (0.34)	0.05^c^ (0.02)	0.01^b^ (0.04)	5.34^c^ (0.47)	0.31^c^ (0.03)	0.03^b^ (0.02)
LSD at 0.05	1.59	0.02	0.01	1.15	0.09	0.03
**pH**						
Control	6.86^a^ (0.16)	0.07^a^ (0.02)	0.01 (0.00)	10.21^b^ (0.51)	0.63^ab^ (0.02)	0.10^a^ (0.2)
5	6.39^ab^ (0.17)	0.07^a^ (0.01)	0.01 (0.00)	12.61^a^ (0.86)	0.70^a^ (0.04)	0.10^a^ (0.01)
6	6.45^ab^ (0.72)	0.05^bc^ (0.02)	0.01 (0.00)	10.03^b^ (0.96)	0.62^ab^ (0.05)	0.09^a^ (0.03)
7	6.55^ab^ (0.25)	0.06^b^ (0.04)	0.01 (0.00)	9.48^b^ (0.65)	0.58^bc^ (0.02)	0.09^a^ (0.01)
8	5.91^ab^ (0.69)	0.05^bc^ (0.02)	0.01 (0.00)	7.25^c^ (0.37)	0.50^cd^ (0.00)	0.07^b^ (0.00)
9	5.17^bc^ (0.20)	0.04^c^ (0.00)	0.01 (0.00)	5.69^c^ (0.41)	0.44^d^ (0.01)	0.05^c^ (0.00)
10	3.77^c^ (0.12)	0.02^d^ (0.01)	0.01 (0.00)	3.40^d^ (0.19)	0.33^e^ (0.00)	0.03^d^ (0.00)
LSD at 0.05	1.30	0.01	NS	1.90	0.09	0.02
**NaCl concentration (mM)**						
Control	8.19^a^ (0.05)	0.08^a^ (0.02)	0.04^a^ (0.01)	9.94^a^ (0.16)	0.59^a^ (0.02)	0.09^a^ (0.00)
50	5.90^c^ (0.07)	0.06^c^ (0.04)	0.03^a^ (0.02)	8.38^bc^ (0.21)	0.55^ab^ (0.01)	0.07^c^ (0.00)
100	7.23^b^ (0.10)	0.07^b^ (0.01)	0.02^ab^ (0.00)	8.97^ab^ (0.29)	0.56^a^ (0.00)	0.08^b^ (0.00)
150	6.23^c^ (0.06)	0.06^c^ (0.00)	0.02^ab^ (0.00)	9.28^a^ (0.24)	0.57^a^ (0.01)	0.07^c^ (0.03)
200	5.71^c^ (0.07)	0.05^d^ (0.00)	0.01^ab^ (0.01)	8.20^c^ (0.05)	0.51^b^ (0.00)	0.07^c^ (0.02)
250	5.53^c^ (0.58)	0.05^d^ (0.03)	0.01^ab^ (0.02)	8.70^abc^ (0.00)	0.55^a^ (0.02)	0.06^d^ (0.01)
300	NG	NG	NG	NG	NG	NG
LSD at 0.05	0.73	0.01	0.02	0.75	0.03	0.01
**Osmotic potential (MPa)**						
Control	6.19^a^ (0.45)	0.07^a^ (0.00)	0.02^a^ (0.00)	8.91^a^ (0.35)	0.63^a^ (0.02)	0.09^a^ (0.00)
-0.2	5.92^a^ (0.19)	0.05^b^ (0.00)	0.01^b^ (0.00)	7.48^a^ (0.49)	0.52^b^ (0.03)	0.07^b^ (0.00)
-0.4	1.53^b^ (0.40)	0.01^d^ (0.00)	0.001^c^ (0.00)	3.50^bc^ (0.88)	0.32^c^ (0.05)	0.02^d^ (0.01)
-0.6	2.32^b^ (0.09)	0.02^c^ (0.00)	0.001^c^ (0.00)	4.86^b^ (0.13)	0.35^c^ (0.01)	0.04^c^ (0.00)
-0.8	NG	NG	NG	NG	NG	NG
-1.0	NG	NG	NG	NG	NG	NG
HSD at 0.05	0.99	0.01	0.003	1.67	0.10	0.02
**Seed burial depths (cm)**						
0	NG	NG	NG	NG	NG	NG
1	4.28^a^ (0.14)	0.04^a^ (0.00)	0.01 (0.00)	5.17^a^ (0.12)	0.38^a^ (0.03)	0.04^a^ (0.00)
2	4.39^a^ (0.08)	0.03^b^ (0.00)	0.01 (0.00)	4.95^a^ (0.08)	0.34^a^ (0.02)	0.04^a^ (0.00)
3	4.16^b^ (0.05)	0.03^b^ (0.00)	0.01 (0.00)	4.76^ab^ (0.09)	0.27^b^ (0.03)	0.03^b^ (0.00)
4	3.93^c^ (0.09)	0.03^b^ (0.00)	0.01 (0.00)	3.68^b^ (0.34)	0.24^bc^ (0.03)	0.03^b^ (0.00)
5	2.10^d^ (0.06)	0.02^c^ (0.00)	0.01 (0.00)	2.36^c^ (0.05)	0.15^c^ (0.02)	0.01^c^ (0.00)
6	NE	NE	NE	NE	NE	NE
7	NE	NE	NE	NE	NE	NE
LSD at 0.05	0. 12	0.01	NS	0. 42	0.06	0.01

NG or NE, no germination or emergence. The values within the column followed by different letters were significantly different at *P* ≤ 0.05. LSD = Least significant difference. Values in parentheses are standard errors of the mean.

Among all the temperatures, the length, fresh and dry weight of root and shoot was maximum at 25 °C followed by 20 °C temperature and minimum values of these parameters were observed at 35 °C (Table 2).

### Impact of pH

**Figure 2:**
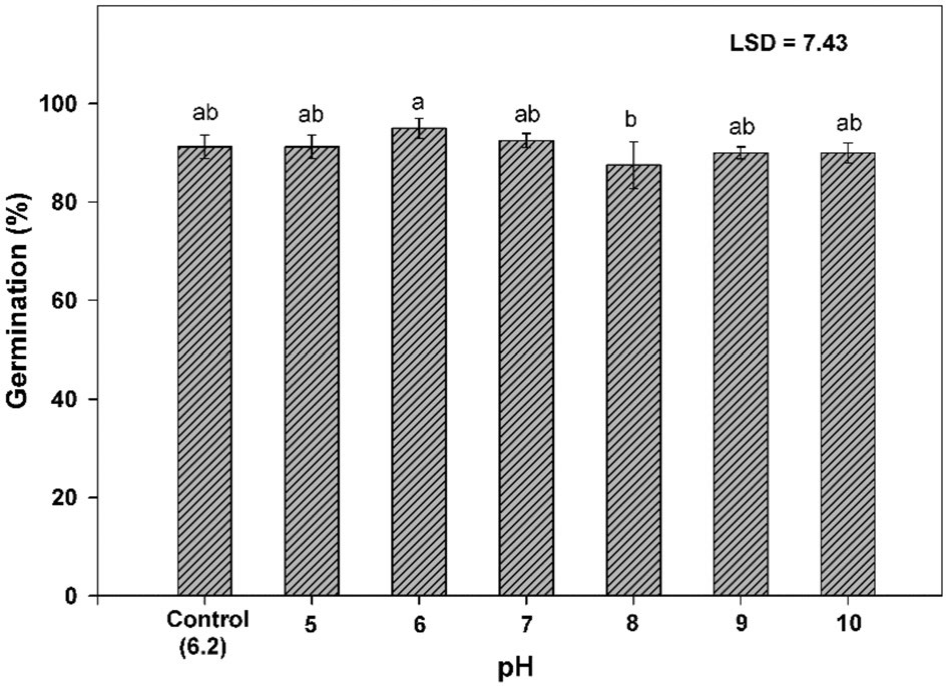
Influence of pH on germination of perennial ryegrass. Nails on the vertical bars represent standard error of the means.

The pH did not affect seed germination of perennial ryegrass and germination remained more than 87% within pH range of 5 to 10 (Fig. 2).

The higher seed germination of perennial ryegrass under a varied range of pH showed that pH would not be a limiting factor for its germination. However, the highest and lowest seed germination was recorded at pH 6 and 8, respectively (Fig. 2). The shortest Ti was observed at pH 7 and control (pH 6.2) (Table 1). The lowest and highest *T*_50_ were measured at the control and pH 8, respectively, however, a similar trend was also observed for *MGT.*

Data in Table 2 specified that maximum root length and fresh weight were attained at control (pH 6.2). The root dry weight was non-significant. There was no deviation in root length of perennial ryegrass from pH 5–8. The minimum values of root length and fresh weight were recorded at pH 10. However, the peak value of shoot length, fresh weight and dry weight of perennial ryegrass were recorded at pH 5 (Table 2).

### Impact of NaCl

**Figure 3:**
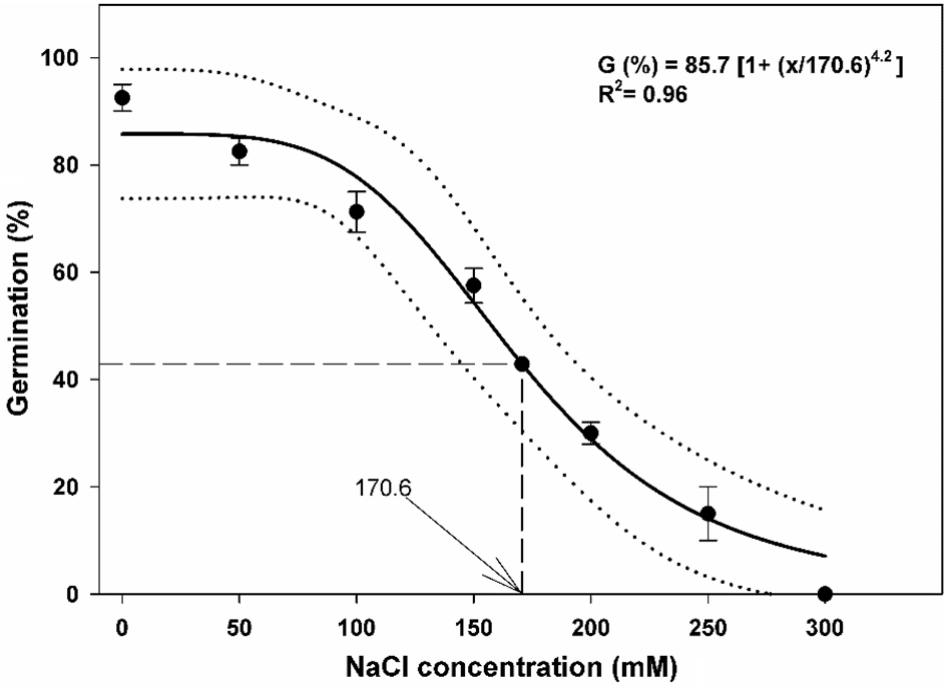
Influence of NaCl concentration on seed germination of perennial ryegrass. Bold line represents a three-parameter logistic model (G (%) = G_max_/[1 + (x/x_50_)^g^]) fitted to the data. The vertical dash line represents X-axis value at 50% of the maximum germination. Dotted liens show 95% confidence intervals. Vertical bars represent ± standard error of the mean.

A 3-parameter sigmoid model (G (%) = 85.7 [1 + (x/170.6)^4.2^], R^2^ = 0.96) was fixed to the germination data of perennial ryegrass in response to various concentrations of NaCl (Fig. 3). The model showed that seed germination was reduced linearly with a rise in NaCl concentration from 0 to 250 mM. The highest seed germination was recorded where no salt stress was applied. Only 15% germination was observed at 250 mM NaCl concentration.

No germination was seen at 300 mM NaCl concentration (Fig. 3). The model estimated that 50% germination of the maximum occurred at 170.6 mM NaCl concentration. Compared to control, *Ti* of perennial ryegrass was postponed nearly to 1 day at 50, 100, 150, 200 and nearly to 5 days at 250 mM NaCl concentration (Table 1). The *T*_50_ and *MGT* were greater in control (distilled water) as compared to each NaCl concentration, showing less germination of perennial ryegrass in reply to increasing concentration of NaCl (Table 1). For the rate of germination, *GI* was higher in control than all other concentrations of NaCl used. The seedling growth parameters of *L. perenne* indicated that by increasing salt stress the fresh weight, dry weight and length of root and shoot decreased significantly (Table 2). Compared to all NaCl concentrations, the highest perennial ryegrass fresh and dry weight of root as well as root and shoot length was recorded in control (distilled water) (Table 2).

### Impact of osmotic potential

Seed germination percentage decreased with an increase in osmotic potential. The seed germination was decreased from 92 to 27% when the osmotic potential was decreased from 0 to − 0.6 MPa.

**Figure 4:**
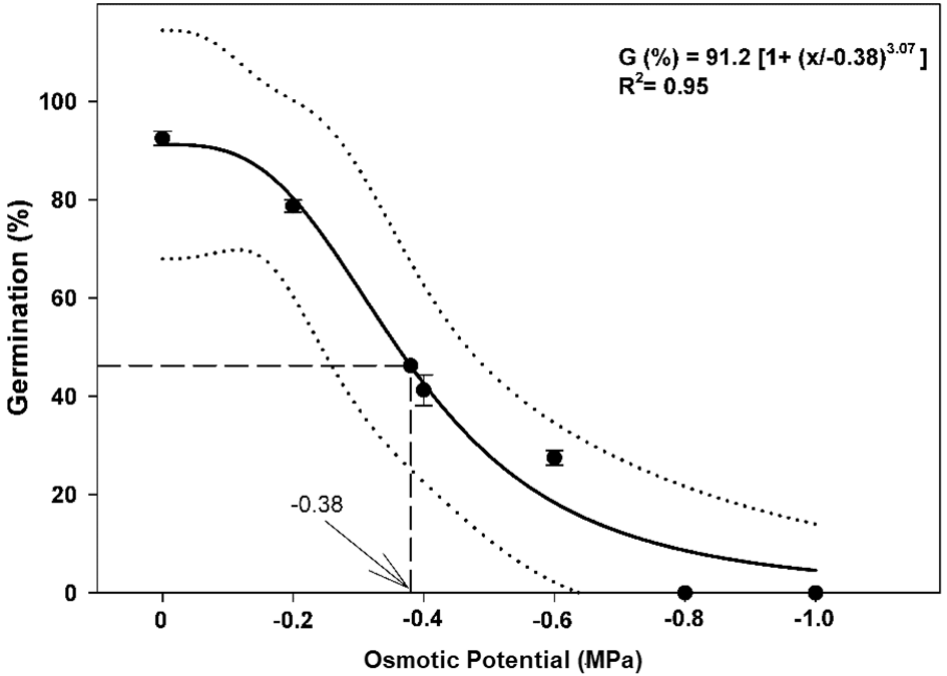
Influence of osmotic potential on seed germination of perennial ryegrass. Bold line represents a three-parameter logistic model (G (%) = G_max_/[1 + (x/x_50_)^g^]) fitted to the data. Vertical dash line represents X-axis value at 50% of the maximum germination. Dotted liens show 95% confidence intervals. Vertical bars represent ± standard error of the mean.

No germination was observed where osmotic potential − 0.8 to − 1.0 was applied. The model estimated that 50% germination of the maximum was obtained at − 0.38 MPa (Fig. 4). The *Ti*, *T*_50_ and *MGT* of perennial ryegrass were delayed with decreasing osmotic potential (Table 1). Compared to distilled water, *Ti*, *T*_50_ and *MGT* were delayed nearly to 2–4 days. The maximum *GI* was recorded with distilled water, whilst it decreased when the osmotic potential was reduced from 0 to − 0.6 MPa (Table 1). In the case of seedling growth, data showed that decrease in osmotic potential from 0 to − 0.6 MPa, and the seedling growth parameters were reduced. The highest root length, fresh weight, and dry weight of perennial ryegrass were observed in distilled water. The shoot length, and fresh and dry weight of perennial ryegrass were also maximum in distilled water and the lowest values of these parameters were recorded at − 0.4 MPa (Table 2).

### Impact of seed burial depth

No seedling emergence was observed when seeds were placed at the soil surface. The seed emergence was reduced linearly from 1 to 5 cm and inhibited completely at 6 or 7 cm burial depth.

**Figure 5:**
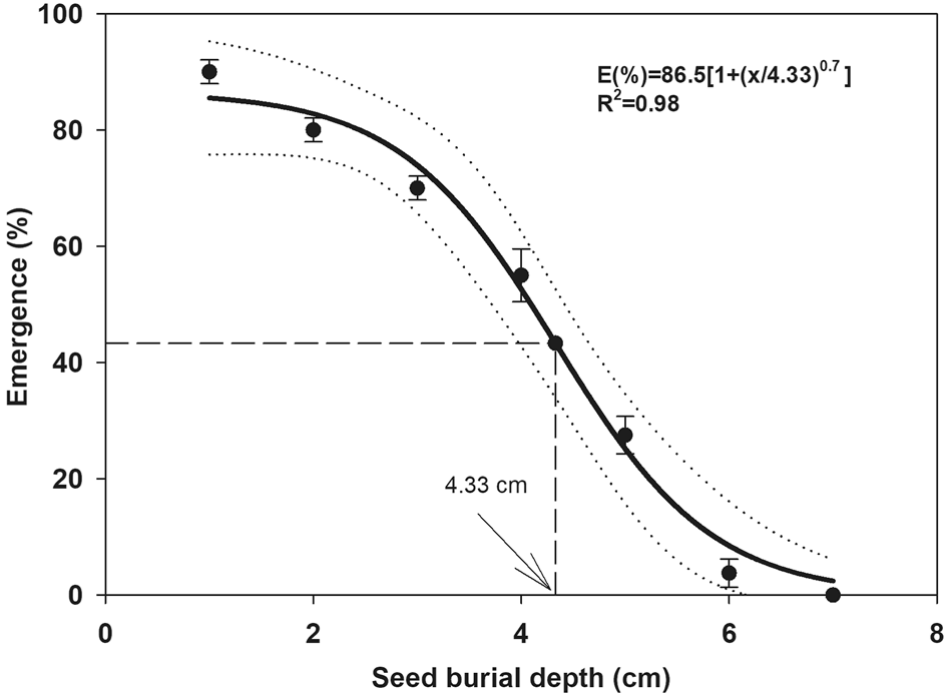
Influence of seed burial depth on seed emergence of perennial ryegrass.. Bold line represents a three-parameter logistic model (E (%) = E_max_/[1 + (x/x_50_)^g^]) fitted to the data. The vertical dash line represents X-axis value at 50% of the maximum germination. Dotted liens show 95% confidence intervals. Vertical bars represent ± standard error of the mean. No emergence was recorded at 0 cm seed burial depth so, the data of 0 cm were not included in the fitted model.

The maximum seedling emergence occurred at 1 cm depth and declined quickly to 55 and 28% at 4 and 5 cm seedling depths, respectively. The model estimated that 50% emergence of the maximum was obtained at 4.33 cm seed burial depth (Fig. 5). The seed emergence parameters such as *Ti*, *E*_50_, *MET* were directly proportional to seed burial depth (Table 1). At 1 or 2 cm depth, seed emergence started in 3.2 and 3.7 days, respectively; however, with increasing burial depth from 3 to 5 cm, *Ti* was delayed by 1 to 3 days. The *E*_50_ and *MET* were minimum at 1 cm and maximum at 5 cm seed burial depth (Table 1). The emergence index (*EI*) was reduced with increasing seed burial depth. The seed placed at 1 cm depth recorded the highest value (3.5) of *El* that gradually decreased to 1.3 and 0.6 at 4 and 5 cm burial depths, respectively. The seedling growth of perennial ryegrass presented in Table 2 indicated that maximum root and shoot length were observed at 2 cm burial depth. The highest fresh and dry weight of root and shoot were also recorded at the same seeding depth (Table 2).

## Discussion

### Impact of temperature

The temperature has a major influence on the occurrence and speed of Lolium species germination by affecting the seed deterioration, loss of dormancy and germination processes^27^. Our data showed that the optimum temperature for maximum germination of perennial ryegrass is 25 °C. It is the winter season weed and the temperature in November and December is about 25 °C. During these months, this weed has the ability to spread in different localities. The low germination at 35 °C may have biological significance because, in field conditions, soil warms slowly, resulting in significant germination causing a problem for growers^28^. Previous work showed that perennial ryegrass germination was typically reduced below a soil temperature of 15 °C, however, low temperature has little influence on the potential survival of seeds^29^. The germination rate parameters like *Ti*, *T*_50_ and *MGT* were lower and the germination index was higher at 25 °C, indicating the most suitable temperature for the germination requirement of this weed. Germination of perennial ryegrass was started on the second day at 25 °C and prolonged to four days with an increase of decrease in temperature. According to the Association of Official Seed Analysts^30^, the first and final counts of germination should take place on day 5 and 14 for most of the *Lolium* species, however, germination may have been delayed to 14 days at low-temperature regimes. Root and shoot length, fresh and dry weight were also maximum at 25 °C. Root length and shoot length varied with varying temperatures because temperature affected the growth and development of plants beyond optimum temperature^31^.

### Impact of pH

Our results indicated that germination of perennial ryegrass was not affected by pH and it can grow on a wide range of pH from 5 to 10. Rather than the addition of NaOH or HCl to water, we used MES, HEPES and TRICINE for the perpetration of desired pH buffer to avoid change in pH during the experiment. It is notable that although different buffers have different chemicals, have no significant effects on the germination of perennial ryegrass*.* According to Thomas et al.^32^, many weed species germinate over a wide range of pH from 6 to 9 but some species showed the problem in germination when exposed to an acidic range (pH 5–6)^33,34^. Germination at high pH suggested that germination of this species will be inhibited under CO_3_^2−^ and HCO_3_^−^ salts. Likewise, germination, the values of germination parameters over time, *T*_50_, and *MGT* of perennial ryegrass were not affected by pH ranges For seedling parameters of perennial ryegrass*,* our findings are parallel to those of Chejara et al.^33^ who reported that some species showed minute differences in root and shoot length over the wide ranges of pH. Our results showed that pH has a negative correlation with shoot length might be due to the increased concentration of salts with an increasing pH value. The ability to germinate over a wide range of pH buffers (5–10) may not provide a reliable indicator of the effects of alkaline or acidic soil on seed germination.

### Impact of salinity

Salinity decreased germination rate progressively with an increase in concentration, suggesting that this species may not be established well in highly saline areas. It is documented that a higher concentration of NaCl can inhibit the germination of many weed species and may die at some stages^6^. In contrast, some weed species are resistant to NaCl concentration and can germinate successfully under salinity stress^9^. In the case of perennial ryegrass*,* the germination percentage, *Ti*, *T*_50_, and *MGT* were reduced with an increase in salt concentration might be due to less water uptake and providing suitable environments for the entrance of noxious ions in the embryo. According to Chachalis et al.^35^, seed germination was significantly affected by salinity and may generate low water potential and provide suitable environments for the access of noxious ions to the embryo. Higher NaCl concentration (300 mM) reduced the germination index and *Ti* which ultimately reduced the germination capability of many weed seeds^36^. Similar to our results for *GI,* Tanveer et al.^34^ concluded that *GI* of *Cucumis melo* was maximum at a lower salinity level. The results suggested that salt stress caused a reduction in root and shoot growth parameters of perennial ryegrass*,* which might be due to less water uptake under high concentrations. Salinity decreased the ease with which seeds take up moisture or facilitated the intake of toxic ions, leading to changes in hormonal and enzymatic activity of seeds and resulting in inhibition in seed germination. An example of the first mechanism, Prisco et al.^37^ found that germination of red kidney bean (*Phaseolus vulgaris* L.) was inhibited by the osmotic effect of NaCl at greeter than − 0.8 MPa osmotic potential. Evidence of the second mechanism was described by Kim and Park^38^ who found salinity reduced the biosynthesis of gibberellic acid, an essential hormone for breaking dormancy and controlling the growth of seedlings. Resultantly, salinity might affect some physiological processes in plants but it is unclear whether the reduction in germination is directly due to osmotic effects, or to some non-toxic germination repression caused by the salt.

### Impact of osmotic potential

The seed germination of perennial ryegrass was decreased by increasing osmotic potential from 0 to − 0.6 and suppressed completely from − 0.8 to − 1 MPa might be due to drought condition that fails the embryo to develop radicle and plumule. The process of water imbibition remains incomplete in drought-sensitive seeds. Dry seeds need more water for cellular metabolism and the amount of water differs from species to species^39^. It has been postulated that metabolites of starch such as glucose are critical for seed germination as they are involved as osmolytes for cellular turgor maintenance and energy sources^40^. Under water-deficit conditions, starch metabolism is reduced, leading to poor germination^41^. Drought affected the *Ti*, *T*_50_, *MGT* and *GI* of perennial ryegrass and the findings of our experiment are similar to the results of Tanveer et al.^42^ who stated that the highest *Ti* (3.54 days) of *Lathyrus aphaca* was measured at the osmotic potential of − 0.6 MPa. In our experiment, fresh and dry weight, as well as shoot and root length of perennial ryegrass, decreased as osmotic potential increased. Contrary to our results, Norsworthy and Oliveira^43^ stated that the shoot length of sicklepod showed resistance to osmotic potential ranges from 0.0 to − 1.0 MPa. The results showed that the osmotic potential of − 0.4 MPa declined the germination of perennial ryegrass up to 40% whereas, the salinity at 100 mM (~ 0.45 MPa) recorded the germination of 80%. This variation in germination might be due to the fact described by Fukuda et al.^44^ that moderate salt levels promote germination and it has been suggested that salt can be compartmentalized and used as cellular osmotica, allowing seeds to germinate under osmotic conditions which would otherwise preclude it. It is further supported by Fukuda et al.^44^ that compartmentalization of salt into vacuoles aid the growth and survival of vegetative plants. Moreover, in the osmotic potential case, the PEG is either unable to cross the cell membrane or can do so only slowly while in the case of salt stress, salt might be compartmentalized and the seed avoids the negative effect of low water potential.

### Impact of seed burial depth

Seeding depth also suppresses the emergence of many weed species^6^. In our study, there was no seed emergence of perennial ryegrass observed at the soil surface, which might be due to a lack of moisture at the soil surface. No germination at the soil surface favored perennial ryegrass weed in the no-till system. These results are in line with those of Javaid and Tanveer^11^ who reported that seeds of *Emex australis* sown on the soil surface had delayed and reduced germination. Emergence was detected at 1 to 5 cmof seed burial depth and maximum emergence was recorded at 1 cmdepth which was probably due to its small seed size and inadequate food reserves, which enabled the seeds to arise from bigger depths. The reduced emergence at greater depth might be due to small amounts of gaseous diffusion in soil and hypoxia^45^. Different weeds germinate to different degrees mainly depending on their seed size and seeding depth^46^. Our study revealed that the deeper the sowing depth of perennial ryegrass*,* the more will be *Ti*, *E*_50_ and *MET.* As deeper the seeds were sown of dove weed, *E*_50_ tended to decline and significantly reduced from 2 to 6 cm^47^. Seed burial depth reduced the *E*_50_ and *MET*^48^. Root and shoot length, and fresh and dry weight of perennial ryegrass decreased as seeding depth increased. Boyd and Van Acker^49^ also reported that the root and shoot fresh weight of numerous weed species decreased with increasing seed burial depth.

## Conclusions

The temperature had a significant impact on the seed germination of perennial ryegrass and the optimum temperature for its germination was 25 °C. It can withstand a wide range of salinity and have the potential to tolerate extreme salinity up to 250 mM NaCl concentration. Osmotic potential reduces its germination and growth beyond − 0.6 MPa and this weed proved to be unaffected by different pH ranges from 5 to 10. It is also revealed that the seed of perennial ryegrass was not germinated at the soil surface, beyond 1–2 cm burial depth a significant reduction was observed and complete inhibition in germination occurred below 5 cm burial depth. The germination and seedling traits were also influenced by these environmental factors.
